# Post-Laser Twin Anemia Polycythemia Sequence: Diagnosis, Management, and Outcome in an International Cohort of 164 Cases

**DOI:** 10.3390/jcm9061759

**Published:** 2020-06-05

**Authors:** Lisanne S.A. Tollenaar, Enrico Lopriore, Stefano Faiola, Mariano Lanna, Julien Stirnemann, Yves Ville, Liesbeth Lewi, Roland Devlieger, Anne Sophie Weingertner, Romain Favre, Sebastian R. Hobson, Greg Ryan, Carlota Rodo, Silvia Arévalo, Philipp Klaritsch, Patrick Greimel, Kurt Hecher, Manuela Tavares de Sousa, Asma Khalil, Basky Thilaganathan, Eric P. Bergh, Ramesha Papanna, Glenn J. Gardener, Andrew Carlin, Elisa Bevilacqua, Victorya A. Sakalo, Kirill V. Kostyukov, Mert O. Bahtiyar, Abigail Wilpers, Mark D. Kilby, Eleonor Tiblad, Dick Oepkes, Johanna M. Middeldorp, Monique C. Haak, Frans J.C.M. Klumper, Joost Akkermans, Femke Slaghekke

**Affiliations:** 1 Department of Obstetrics, Division of Fetal therapy, Leiden University Medical Center, 2333 ZA Leiden, The Netherlands; d.oepkes@lumc.nl (D.O.); j.m.middeldorp@lumc.nl (J.M.M.); m.c.haak@lumc.nl (M.C.H.); f.j.c.m.klumper@lumc.nl (F.J.C.M.K.); j.akkermans@lumc.nl (J.A.); f.slaghekke@lumc.nl (F.S.); 2 Department of Pediatrics, Division of Neonatology, Leiden University Medical Center, 2333 ZA Leiden, The Netherlands; e.lopriore@lumc.nl; 3 Fetal Therapy Unit “U. Nicolini”, Vittore Buzzi Children’s Hospital, University of Milan, 20154 Milan, Italy; info@terapiafetale.it (S.F.); marianomatteo.lanna@fastwebnet.it (M.L.); 4 Department of Obstetrics and Maternal-Fetal Medicine, Hôpital Necker Enfants Malades, AP-HP, 75015 Paris, France; julien.stirnemann@nck.aphp.fr (J.S.); ville.yves@gmail.com (Y.V.); 5 Department of Obstetrics and Gynecology, University Hospitals Leuven, 3000 Leuven, Belgium; liesbeth.lewi@uzleuven.be (L.L.); roland.devlieger@uzleuven.be (R.D.); 6 Department of Obstetrics and Gynecology, Strasbourg University Hospital, CEDEX, 67000 Strasbourg, France; anne-sophie.weingertner@chru-strasbourg.fr (A.S.W.); romain.favre@chru-strasbourg.fr (R.F.); 7 Fetal Medicine Unit, Department of Obstetrics & Gynecology, Mount Sinai Hospital, University of Toronto, Toronto, ON M5G 1X5, Canada; Sebastian.Hobson@sinaihealthsystem.ca (S.R.H.); Greg.Ryan@sinaihealth.ca (G.R.); 8 Maternal Fetal Medicine Unit, Department of Obstetrics, Vall d’Hebron University Hospital, 08035 Barcelona, Spain; crodo@vhebron.net (C.R.); siareval@vhebron.net (S.A.); 9 Division of Obstetrics and Maternal Fetal Medicine, Department of Obstetrics and Gynecology, Medical University of Graz, 8036 Graz, Austria; philipp.klaritsch@medunigraz.at (P.K.); patrick.greimel@medunigraz.at (P.G.); 10 Department of Obstetrics and Fetal Medicine, University Medical Center Hamburg-Eppendorf, 20251 Hamburg, Germany; k.hecher@uke.de (K.H.); m.tavares-de-sousa@uke.de (M.T.d.S.); 11 Fetal Medicine Unit, St George University Hospital NHS Foundation Trust, London SW17 0RE, UK; asmakhalil79@googlemail.com (A.K.); baskaran.thilaganathan@stgeorges.nhs.uk (B.T.); 12 The Fetal Center, Department of Obstetrics, Children’s Memorial Hermann Hospital, Gynecology and Reproductive Sciences, UT Health, McGovern Medical School, University of Texas, Houston, TX 77030, USA; Eric.P.Bergh@uth.tmc.edu (E.P.B.); ramesha.papanna@uth.tmc.edu (R.P.); 13 Department of Maternal Fetal Medicine, Mater Mothers’ Hospital, South Brisbane, QLD 4101, Australia; glenn.gardener@mater.org.au; 14 Department of Obstetrics and Gynecology, University Hospital Brugmann, Université Libre de Bruxelles, 1020 Brussels, Belgium; andrew.carlin@chu-brugmann.be (A.C.); elisa.bevilacqua@chu-brugmann.be (E.B.); 15 Acad. V.I. Kulakov Research Center of Obstetrics, Gynecology, and Perinatology, Ministry of Health of the Russian Federation, 495 Moscow, Russia; v_sakalo@oparina4.ru (V.A.S.); kostyukov_k@yahoo.com (K.V.K.); 16 Department of Obstetrics, Gynecology and Reproductive Sciences, Yale School of Medicine, New Haven, CT 06510, USA; mert.bahtiyar@yale.edu (M.O.B.); abigail.wilpers@yale.edu (A.W.); 17 Fetal Medicine Centre, Birmingham Women’s and Children’s Foundation Trust, University of Birmingham, Birmingham B4 6NH, UK; M.D.KILBY@bham.ac.uk; 18 Center for Fetal Medicine, Karolinska University Hospital, 171 76 Stockholm, Sweden; eleonor.tiblad@sll.se

**Keywords:** monochorionic twins, twin anemia polycythemia sequence, TAPS, twin-twin transfusion syndrome, TTTS, laser surgery, management, perinatal mortality, fetal demise, neonatal morbidity

## Abstract

The aim of this study was to investigate the management and outcome in the post-laser twin anemia polycythemia sequence (TAPS). Data of the international TAPS Registry, collected between 2014 and 2019, were used for this study. The primary outcomes were perinatal mortality and severe neonatal morbidity. Secondary outcomes included a risk factor analysis for perinatal mortality and severe neonatal morbidity. A total of 164 post-laser TAPS pregnancies were included, of which 92% (151/164) were diagnosed antenatally and 8% (13/164) postnatally. The median number of days between laser for TTTS and detection of TAPS was 14 (IQR: 7–28, range: 1–119). Antenatal management included expectant management in 43% (62/151), intrauterine transfusion with or without partial exchange transfusion in 29% (44/151), repeated laser surgery in 15% (24/151), selective feticide in 7% (11/151), delivery in 6% (9/151), and termination of pregnancy in 1% (1/151). The median gestational age (GA) at birth was 31.7 weeks (IQR: 28.6–33.7; range: 19.0–41.3). The perinatal mortality rate was 25% (83/327) for the total group, 37% (61/164) for donors, and 14% (22/163) for recipients (*p* < 0.001). Severe neonatal morbidity was detected in 40% (105/263) of the cohort and was similar for donors (43%; 51/118) and recipients (37%; 54/145), *p* = 0.568. Independent risk factors for spontaneous perinatal mortality were antenatal TAPS Stage 4 (OR = 3.4, 95%CI 1.4-26.0, *p* = 0.015), TAPS donor status (OR = 4.2, 95%CI 2.1–8.3, *p* < 0.001), and GA at birth (OR = 0.8, 95%CI 0.7–0.9, *p* = 0.001). Severe neonatal morbidity was significantly associated with GA at birth (OR = 1.5, 95%CI 1.3–1.7, *p* < 0.001). In conclusion, post-laser TAPS most often occurs within one month after laser for TTTS, but may develop up to 17 weeks after initial surgery. Management is mostly expectant, but varies greatly, highlighting the lack of consensus on the optimal treatment and heterogeneity of the condition. Perinatal outcome is poor, particularly due to the high rate of perinatal mortality in donor twins.

## 1. Introduction

The twin anemia polycythemia sequence (TAPS) can arise from chronic unbalanced feto-fetal transfusion through minuscule placental anastomoses in monochorionic twins, leading to anemia in the TAPS donor and polycythemia in the TAPS recipient [1]. Unlike twin-twin transfusion syndrome (TTTS), TAPS develops in the absence of the twin oligohydramnios-polyhydramnios sequence. The iatrogenic form of TAPS, post-laser TAPS, can develop in 2–16% after laser surgery for TTTS due to the presence of minuscule residual anastomoses [2,3,4]. The rate of post-laser TAPS can be reduced by using the Solomon technique instead of the selective laser technique (3% vs. 16%, respectively) [2]. With this approach, the entire placental vascular equator is laser photocoagulated, thereby blocking all anastomoses, even the miniscule ones that may not be visualized. Although the rate of residual anastomoses has significantly dropped after the implementation of the Solomon technique, post-laser TAPS may still occur [2,5,6]. Options to manage post-laser TAPS include expectant management, preterm delivery, intrauterine transfusion (IUT) with or without a partial exchange transfusion (PET), fetoscopic laser surgery, and selective feticide [7]. The best treatment has not been established. The technical feasibility of a second intrauterine intervention may be limited due to complications that have arisen from the initial laser procedure for TTTS, such as amnion-chorion separation or preterm premature rupture of the membranes (PPROM). For informed decision making regarding the preferred intervention, it is crucial to have insight into perinatal outcome of post-laser TAPS twins. Due to the rarity of the complication, perinatal outcome is insufficiently investigated, and available information is based on small cohort studies. To expand our knowledge on TAPS, we set up the TAPS Registry, an international collaboration aimed at collecting data on diagnosis, treatment, and outcome in TAPS twins.

In the current study, the data from this TAPS Registry were used to (1) characterize diagnosis, treatment modalities, and outcome in post-laser TAPS twins, (2) to compare perinatal outcome between TAPS donors and recipients, and (3) to investigate potential risk factors for adverse perinatal outcome.

## 2. Experimental Section

The TAPS Registry, established in 2014, was a web-based registry for anonymous data collection (www.tapsregistry.org). Fetal therapy centers across the world were invited to participate. Participating centers were provided with personal credentials to enter the data of their TAPS cases into the online registry. Between 2014 and 2019, seventeen specialized fetal therapy centers contributed to data collection (see Appendix A).

All monochorionic twin pregnancies diagnosed with post-laser TAPS were considered eligible for this study. Pregnancies with spontaneous TAPS were excluded and were described in a separate study [8].

Antenatal diagnosis of TAPS was based on discordant middle cerebral artery peak systolic velocity (MCA-PSV) measures, with an increased MCA-PSV (>1.5 multiples of the median (MoM)) in the TAPS donor, indicative of fetal anemia, combined with a decreased MCA-PSV measure (<1.0 MoM) in the TAPS recipient, indicative of fetal polycythemia [9]. Postnatal diagnosis was reached by the presence of an inter-twin hemoglobin difference > 8.0 g/dL combined with at least one of the following: a reticulocyte count ratio > 1.7 or the presence of only minuscule vascular anastomoses (diameter < 1 mm) detected through color dye injection of the placenta [10,11]. Cases that were diagnosed with TAPS within one week after laser surgery for TTTS were excluded from the study, unless TAPS persisted. We did this as a large MCA-PSV discrepancy shortly after laser is likely to be a result of hemodynamic re-equilibration and is not based on TAPS [12].

The following information was obtained from local medical records: gravidity, parity, location of the placenta, moment of diagnosis (antenatal or postnatal), gestational age (GA) at diagnosis, TAPS stage at diagnosis, and the presence of additional ultrasound findings including "*starry-sky liver”* in the recipient and/or a difference in placental echogenicity. The severity of antenatal TAPS was determined according to the previously published staging system by Slaghekke et al. [13]. For post-laser TAPS specifically, the following data regarding preceding TTTS were collected: Quintero stage, GA at laser, laser technique, operator’s opinion on completeness of the laser procedure, and TTTS-donor-recipient role. In addition, the antenatal management for TAPS was recorded, including expectant management, delivery (defined as a delivery within 7 days after diagnosis), IUT (± PET), fetoscopic laser surgery, selective feticide, and termination of pregnancy (TOP). Furthermore, information on placental color dye injection was collected, including classification of the type (arterio-venous (AV), veno-arterial (VA), arterio-arterial (AA), veno-venous (VV)), and the number and size of anastomoses. Perinatal outcome measures included: TAPS donor/recipient status, birth weight, hemoglobin and reticulocyte values, treatment with blood transfusion or partial exchange transfusion on Day 1, the presence of severe neonatal morbidity and/or severe cerebral injury, and the occurrence of perinatal mortality.

Primary outcomes were perinatal mortality and severe neonatal morbidity. Perinatal mortality was defined as fetal demise or neonatal death within 28 days after birth. In the context of selective feticide or TOP, a distinction was made between spontaneous fetal demise and intended fetal demise. Severe neonatal morbidity was defined as the presence of at least one of the following, diagnosed within 28 days after birth or prior to discharge: respiratory distress syndrome requiring mechanical ventilation and surfactant, patent ductus arteriosus requiring treatment, necrotizing enterocolitis ≥ Stage 2 [14], retinopathy of prematurity ≥ Stage 3 [15], amniotic band syndrome, ischemic limb injury, or severe cerebral injury. Severe cerebral injury was diagnosed in case of one of the following abnormalities was identified on cerebral imaging: intraventricular hemorrhage ≥ Stage 3 [16], ventricular dilatation (including post-hemorrhagic ventricular dilatation) [17], cystic periventricular leukomalacia ≥ Grade 2 [18], porencephalic or parenchymal cysts, arterial infarction, or other severe cerebral lesions associated with an adverse outcome.

Secondary outcomes included diagnosis- and therapy-related characteristics, hematological and placental characteristics, and a risk factor analysis for spontaneous perinatal mortality and severe neonatal morbidity. Cases with intended fetal demise in the context of selective feticide or termination of pregnancy were excluded for the risk factor analysis for spontaneous perinatal mortality. Since TAPS cases may be managed according to different strategies in one pregnancy, management-group assignment was based on the first treatment that was performed. The following parameters were investigated in the univariate risk analysis: GA at diagnosis of TAPS, GA at laser for TTTS, days between laser for TTTS and development of TAPS, Quintero stage, antenatal TAPS stage, persistence of TTTS-TAPS donor-recipient status, type of antenatal management, and GA at birth (in weeks). For antenatal TAPS stage, the highest recorded antenatal TAPS stage was selected. In case of TAPS Stage 5, the highest TAPS stage before Stage 5 was used. For the risk factor analysis for severe neonatal morbidity, two more parameters were added: severe growth restriction defined as birth weight < 3rd centile and the presence of postnatal TAPS.

The following additional outcomes were determined: inter-twin hemoglobin difference (highest hemoglobin value–lowest hemoglobin value), reticulocyte count ratio (highest reticulocyte value (‰)/lowest reticulocyte value (‰)), the presence of severe growth restriction (defined as a birth weight < 3rd centile according to Hoftiezer [19]), postnatal TAPS stage (according to Slaghekke [7]), and the configuration of anastomosis type per TAPS placenta.

Statistical analyses were carried out using SPSS Version 25.0 (IBM, Armonk, NY, USA). Data are presented as the mean ± standard deviation (SD) or as median and interquartile range (IQR) and/or range (minimum-maximum), as appropriate. A *p*-value < 0.05 was considered statistically significant. Differences between donors and recipients were calculated using the paired *t*-test for normally distributed continuous outcomes. To account for the fact that observations between co-twins are not independent, the Generalized Estimated Equation module was executed for analyses per fetus or neonate. Potential risk factors were checked for correlation using Spearman’s rank test (R). A correlation coefficient R > (−) 0.7 was considered to indicate a strong relationship between the factors. Potential risk factors for perinatal mortality and severe neonatal morbidity were assessed in a univariate logistic regression model. A multivariate logistic regression model was applied to the variables that showed significant association in the univariate analysis. Results are expressed as odds ratios (OR) with 95% confidence intervals (CI).

## 3. Results

Of the 422 TAPS cases entered into the TAPS Registry, two-hundred forty-nine (59%) were spontaneous TAPS and were excluded from the study. The remaining 173 (41%) were post-laser TAPS and eligible for the study. In eight post-laser TAPS cases, TAPS was diagnosed within one week after laser for TTTS. As TAPS did not persist one week after laser surgery for TTTS, these cases were excluded. One case was excluded based on TAPS being diagnosed at Stage 5. A total of 164 post-laser TAPS cases were included in the analysis for the current study.

**Figure 1:**
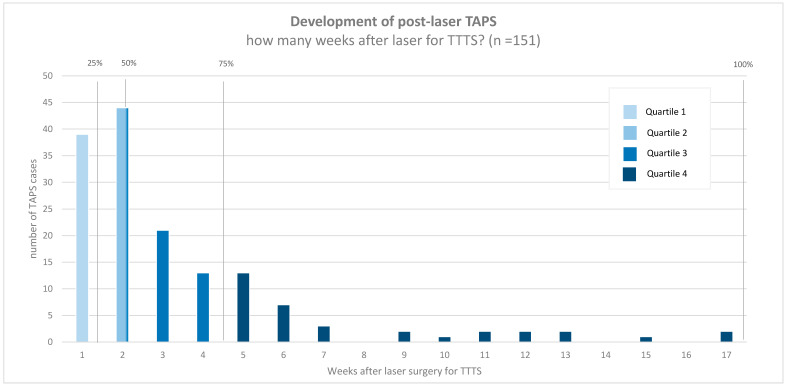
Weeks between fetoscopic laser surgery for twin-twin transfusion syndrome and diagnosis of post-laser twin anemia polycythemia sequence.

TAPS was diagnosed antenatally in 92% (151/164) and only postnatally in 8% (13/164) of the group (Table 1). Laser for TTTS (prior to post-laser TAPS) was performed using the Solomon technique in 37% (60/164) and the selective technique in 63% (104/164). The operating surgeon assumed that the laser for TTTS was complete in 81% (126/156) of post-laser TAPS cases, in 74% (43/58) of cases treated with the Solomon technique, and in 85% (83/98) of cases treated with the selective technique. Reasons for incomplete laser were: poor visibility (*n* = 11), fetal position (*n* = 7), placental position (*n* = 5), bleeding (*n* = 2), velamentous anastomoses (*n* = 1), vomiting of patient (*n* = 1), and not specified (*n* = 3). In half of the antenatally detected TAPS cases, TAPS developed within 14 days (IQR: 7–28; range 1–119) after laser treatment for TTTS. Figure 1 depicts time between laser for TTTS and the diagnosis of post-laser TAPS for all antenatally diagnosed cases. In 45% (73/161) of cases, TTTS donors became TAPS donors. Antenatal management included expectant management in 43% (62/151), IUT (± PET) in 29% (44/151), laser reintervention in 15% (24/151), selective feticide in 7% (11/151), delivery in 6% (9/151), and TOP in 1% (1/151) (Table 2).

In total, 51% (84/164) of post-laser TAPS placentas were injected with color dye (Table 3). Of the 84 injected placentas, ten cases were treated with laser reintervention, and 74 cases were not treated with laser reintervention. In placentas not treated with laser reintervention, the median total number of anastomoses was one (IQR: 0–1), and 55% (40/74) had only AV anastomoses running in one direction. AA and VV anastomoses were detected in 8% (6/74) and 7% (5/74) of the group, respectively. In ten TAPS placentas, no residual anastomoses were found after color dye injection: five placentas belonged to cases with spontaneous resolution of TAPS after expectant management or treatment with IUT (±PET), and the other five belonged to cases with TAPS confirmed both antenatally and postnatally. Residual anastomoses were found in 40% (4/10) of post-laser TAPS cases treated with laser reintervention. Three cases with residual anastomoses had only one minuscule AV anastomosis, and the fourth had one single minuscule AA anastomosis. Out of the four cases with residual anastomoses, two cases had confirmed postnatal TAPS, one case had a double fetal demise after reintervention with laser, and one case showed an inter-twin hemoglobin difference of 8 g/dL (borderline TAPS) and had a low reticulocyte count ratio, but required a blood transfusion and partial exchange transfusion on day 1.

Table 4 provides information on the perinatal outcome of post-laser TAPS twins, separated for TAPS donors and recipients. Median GA at birth was 31.7 weeks (IQR: 28.6–33.7, range 19.0–41.3). TAPS donors had significantly lower mean birth weights than TAPS recipients, 1346 g ± 525 g vs. 1533 g ± 588 g *p* < 0.001. Fetal demise occurred in 17% (56/327) of the group, either spontaneously in 10% (33/327) or intentionally in 7% (23/327). TAPS donors had a higher risk of fetal demise than TAPS recipients, both for spontaneous fetal demise (15% (25/164) vs. 5% (8/163); *p* < 0.001) and intended fetal demise (11% (18/164) vs, 3% (5/163); *p* = 0.007). The rate of neonatal mortality was 10% (27/271) and was higher in TAPS donors than in TAPS recipients, 15% (18/121) vs. 6% (9/150), respectively (*p* = 0.008). Overall, perinatal mortality (including intended demise) occurred in 25% (83/327) of TAPS twins, in 37% (61/164) of TAPS donors, and 14% (22/163) of TAPS recipients, respectively (*p* < 0.001). Severe neonatal morbidity was diagnosed in 40% (105/263) of liveborn twins and was similar for TAPS donors (43%; 51/118) and TAPS recipients (37%; 54/145), *p* = 0.568. Severe cerebral injury was identified in 11% (28/263) of liveborn twins, in 11% (13/118) of TAPS donors, and in 10% (15/145) of TAPS recipients (*p* = 0.916). Severe cerebral injury was diagnosed based on intraventricular hemorrhage ≥ Grade 3 (*n* = 15), cystic periventricular leukomalacia ≥ Grade 2 (*n* = 5), (post-hemorrhagic) ventricular dilatation (*n* = 6), porencephalic or parenchymal cysts (*n* = 3), arterial infarction (*n* = 1), and other severe lesions associated with adverse outcome (*n* = 1). Ischemic limb injury did not occur in this cohort of post-laser TAPS twins.

In cases that were diagnosed with TAPS at birth (72%; 76/106), the median inter-twin hemoglobin difference was 12.6 g/dL (IQR: 10.3–15.1, range: 8.2–21.7), and the median reticulocyte count ratio was 2.7 (IQR: 2.3–3.9). At birth, 68% (52/76) of donors needed a blood transfusion to treat anemia, and 49% (37/76) of recipients needed a PET to treat polycythemia. In total, 33% (25/76) had Stage 1 postnatal TAPS, 34% (26/76) Stage 2, 20% (15/76) Stage 3, 11% (8/76) Stage 4, and 1% (1/76) Stage 5 postnatal TAPS.

Univariate risk factor analysis showed that spontaneous perinatal mortality was significantly associated with Quintero Stage 3 (OR = 3.6, 95%CI 1.1–12.0, *p* = 0.039), antenatal TAPS Stage 4 (OR = 6.1, 95%CI 1.8–20.4, *p* = 0.003), TAPS donor status (OR = 3.7, 95%CI 2.2–6.3, *p* < 0.001), and GA at birth (OR = 0.8, 95%CI 0.7-1.0, *p* = 0.006). There was no strong correlation between the risk factors (Quintero stage–TAPS donor status (R < 0.001, *p* = 1.000); Quintero stage–antenatal TAPS stage (R = 0.078, *p* = 0.187); TAPS donor status–antenatal TAPS stage (R = −0.005, *p* = 0.931); Quintero stage–GA at birth (R = −0.022, *p* = 0.701); TAPS donor status–GA at birth (R < 0.001, *p* = 0.998); antenatal TAPS stage–GA at birth (R = −0.005, *p* = 0.931)), so all parameters were included in the multivariate analysis. In the multivariate analysis, antenatal TAPS Stage 4 (OR = 6.1, 95%CI 1.4–26.0, *p* = 0.015), TAPS donor status (OR = 4.2, 95%CI 2.1–8.3, *p* < 0.001), and GA at birth (OR = 0.8, 95%CI 0.7–0.9, *p* = 0.001) were identified as independent risk factors for spontaneous perinatal mortality. Univariate risk factor analysis showed that severe neonatal morbidity was significantly associated with GA at birth (OR = 1.5, 95%CI 1.3–1.8, *p* < 0.001) and Quintero Stage 2 (OR = 3.2 95%CI 1.1–9.7, *p* = 0.038). Both parameters were included in multivariate risk factor analysis as no correlation was found between the two (R = −0.022, *p* = 0.701). Multivariate risk factor analysis revealed that only GA at birth was an independent risk factor for severe neonatal morbidity (OR = 1.5, 95%CI 1.3–1.7, *p* < 0.001). More details on the risk analyses for perinatal mortality and severe neonatal morbidity are presented in Table A2 and Table A3 of Appendix B.

## 4. Discussion

This was the first large international study investigating management and outcome in post-laser TAPS twins. Our study showed that post-laser TAPS generally developed within one month after laser for TTTS, but could be detected up to 17 weeks after laser intervention. Management for post-laser TAPS was mostly expectant, but varied considerably, highlighting the lack of consensus for optimal treatment. In this cohort, perinatal outcome was poor, particularly due to high perinatal mortality rates in TAPS donors. This study provided important information for clinicians involved in the care for TTTS twins treated with laser surgery and might contribute to a better understanding of post-laser TAPS.

This is the first study that gives a clear overview of the time of onset of post-laser TAPS and and shows that there is a wide range in timing of presentation. This variation might be attributed to two factors. First, reversal of the donor-recipient role could result in a slower development of post-laser TAPS. Possibly, former TTTS recipients that became TAPS donors may be protected against anemia for a longer period of time due to the excess of blood they received during TTTS. In contrast, former TTTS donors that became TAPS donors might suffer sooner from anemia due to their relatively hypovolemic state. Alternatively, TAPS might develop later in cases with compensating blood supply, allowed by VA, AA, or VV anastomoses. Our results showed that half of the post-laser TAPS cases presented within two weeks after laser surgery for TTTS. Although all cases in this study had signs of ongoing TAPS after the first week, spontaneous normalization of a large MCA-PSV discordancy after laser surgery has also been reported in previous literature [12,20]. Importantly, a large MCA-PSV difference shortly after laser might in some cases be the result of fetal hemodynamic re-equilibration after intervention, rather than the onset of post-laser TAPS due to the presence of a patent anastomosis. Consequently, intervening directly within 1-2 weeks after laser surgery in these cases could lead to unnecessary treatment since there is no ongoing transfusion. Therefore, close follow-up ultrasound examination to identify persistence or progression of a MCA-PSV discrepancy after the first weeks after laser surgery is recommended to confirm the diagnosis of post-laser TAPS and prevent unnecessary intervention and exposure to iatrogenic risks.

Our study demonstrated that 81% of the surgeons initially thought that the laser for TTTS was complete. This “low index of suspicion” causes TAPS to often occur unexpectedly and shows that operator-reported completeness cannot be relied upon. Interestingly, approximately a third of post-laser TAPS twins were treated for TTTS with the Solomon technique. This illustrated that, although the Solomon technique has been proven to decrease the incidence of post-laser TAPS [2], clinicians should remain vigilant for the development of this complication even after a complete Solomon line was thought to be achieved. In agreement with the current recommendations of the twin guideline of the International Society of Ultrasound in Obstetrics and Gynecology [21], we strongly underline the importance of strict routine MCA-PSV Doppler follow-up examination in TTTS twins treated with laser surgery during the entire pregnancy to check for the presence of post-laser TAPS.

We found high rates of perinatal mortality, particularly in the TAPS donor, reflecting the detrimental impact of fetal anemia on perinatal survival. Remarkably, TAPS donors only showed increased risk for perinatal mortality. After birth, donors and recipients had similar rates of severe neonatal morbidity, suggesting that neonatal health is more strongly related to the degree of prematurity than TAPS donor-recipient status. Importantly, this study demonstrated that GA at birth is a strong risk factor for both perinatal mortality and severe neonatal morbidity in post-laser TAPS twins. Compared to post-laser TAPS survivors that were previously investigated in the Solomon trial [22], we reported similar rates of severe neonatal morbidity (38% vs. 39%) and higher rates of perinatal mortality (18% vs. 26%). Of note, long-term outcome was not investigated in this study, and therefore, a difference in long-term neurodevelopmental impairment between donors and recipients cannot be precluded.

Post-laser TAPS twins showed an overall more detrimental outcome than spontaneous TAPS twins [8]. The exact cause of this difference in outcome is not entirely clear, but it is likely to be multifactorial. The first and most obvious explanation is that post-laser TAPS twins have previously experienced TTTS, a severe condition itself [23]. Therefore, their fetal condition might already be compromised when they start developing TAPS. Given the fact that half of the post-laser TAPS cases occur within the first two weeks after laser, twins have had only limited time to recover, making them more prone to decompensation. A second explanation could be found in the angioarchitecture of placentas of post-laser TAPS twins. This study represents the biggest cohort of injected post-laser TAPS placentas and confirms previous findings that post-laser TAPS placentas often show only one or a few placental anastomoses [24]. Additionally, we found that most cases only had unidirectional AV anastomoses, without compensating flow from VA, AA, or VV anastomoses in the opposite direction. This might lead to an accelerated deterioration of TAPS, resulting more rapidly in abnormal Doppler blood flows, hydrops, and fetal death. Interestingly, a minority of post-laser TAPS placentas did not show residual anastomoses, in spite of the presence of confirmed postnatal TAPS. This could be explained by suboptimal color dye or by the presence of deep, hidden anastomoses [4,25]. Third, the choice of antenatal management might have also influenced the condition of post-laser TAPS twins. Our results showed that treatment for post-laser TAPS was diverse, but that the majority of the group was managed expectantly or received IUT (± PET); two treatment strategies that are not definitive in nature and allow the condition to progress. Possibly, laser was considered more challenging or not feasible in cases that already underwent laser, due to expected reduced visibility, membrane separation, iatrogenic monoamnionicity, PPROM, or because of the same reasons that caused the laser to be incomplete in the first place, such as the position of the placenta. Illustratively, we found a high rate (40%) of residual anastomoses in twins treated with laser reintervention. A detailed evaluation of differences in perinatal outcome between the various management strategies will be presented in a separate study [26].

As with all registries, this study was fully dependent on local registrations of post-laser TAPS cases. In many countries, TTTS cases are sent back to the referring hospital after laser procedure, leaving care in less experienced hands. As the diagnosis of post-laser TAPS is only reached by adequate MCA-PSV Doppler examination, hemoglobin and reticulocyte measures, and placental-injection studies, it is likely that some post-laser TAPS cases have been missed. Nonetheless, this study represented the largest cohort of post-laser TAPS twins to date and was able to provide valuable insights into management and outcome in post-laser TAPS.

## 5. Conclusions

To conclude, post-laser TAPS could occur at any time after laser for TTTS, is managed heterogeneously, and is associated with poor outcome, particularly in donor twins. Our findings necessitate further research into the best treatment option for TAPS. To investigate the best treatment for TAPS adequately, an international randomized controlled trial is needed.

## Figures and Tables

**Table 1:** Baseline characteristics for the post-laser twin anemia polycythemia sequence.

	Post-Laser TAPS (*N* = 164 Pregnancies, 328 Fetuses)
Gravidity	2 (1–3)
Parity	0 (0–1)
Antenatal diagnosis of TAPS	151/164 (92)
Location of placenta	
Anterior	72/164 (44)
Posterior	86/164 (52)
Other †	6/164 (4)
TTTS stage ‡	
Q1	20/159 (13)
Q2	75/159 (47)
Q3	59/159 (37)
Q4	5/159 (3)
Solomon technique for TTTS laser	60/164 (37)
GA at laser for TTTS	20.6 (18.0–23.0)
Time between laser and post-laser TAPS (days)	14 (7–28)
Persistence of TTTS-TAPS donor-recipient role §	73/161 (45)
Laser for TTTS complete (surgeon’s opinion) ¶	126/156 (81)

Data are presented as n/N (%) or the median (IQR). † Other types of placental position included: partly anterior and partly posterior (*n* = 1) and partly fundal (*n* = 1) lateral left (*n* = 2) and lateral right (*n* = 2). ‡ Five missing values. § Three missing values. ¶ Eight missing values. TAPS, twin anemia polycythemia sequence; TTTS, twin-twin transfusion syndrome; Q, Quintero; GA, gestational age.

**Table 2:** Diagnosis- and management-related characteristics for the post-laser twin anemia polycythemia sequence.

	Post-Laser TAPS (*N* = 164 Pregnancies, 328 Fetuses)
GA at diagnosis	23.1 (20.4–25.9)
TAPS stage at diagnosis	
1	43/151 (28)
2	69/151 (48)
3	27/151 (17)
4	12/151 (8)
Highest TAPS stage	
1	22/151 (15)
2	62/151(40)
3	26/151 (16)
4	22/151 (14)
5	19/151 (15)
Presence of additional ultrasound markers †	
Starry-sky liver (recipient)	56/141 (40)
Difference in placental echogenicity	35/151 (23)
Antenatal management	
Expectant management	62/151 (43)
Delivery	9/151 (6)
IUT (± PET)	44/151 (29)
Laser surgery	24/151 (15)
Selective feticide	11/151 (7)
Termination of pregnancy	1/151 (1)
Female ‡	162/308 (53)
Cesarean §	206/326 (63)

Data are the median (IQR) or n/N (%). Except for “female” and “cesarean”, all other parameters are presented for antenatally diagnosed post-laser TAPS cases only (*N* = 151).† In 10 cases, the presence of a starry-sky liver was not assessed. ‡ Twenty missing values. § Two missing values.TAPS, twin anemia polycythemia; GA, gestational age; IUT, intrauterine transfusion; PET, partial exchange transfusion.

**Table 3:** Characteristics for placentas not treated with laser reintervention for the post-laser twin anemia polycythemia sequence.

	Injected Post-Laser TAPS Placentas (*N* = 74)
Total number of anastomoses	1 (1–2)
Number of AV anastomoses	1 (0–1)
Number of VA anastomoses	0 (0–1)
Number of AA anastomoses	0 (0–0)
Number of VV anastomoses	0 (0–0)
Presence of anastomoses	
Presence of AV/VA anastomoses	58/74 (80)
Presence of AA anastomoses	6/74 (8)
Presence of VV anastomoses	5/74 (7)
Type of anastomoses per placenta	
No anastomoses	10/74 (14)
AV (one direction)	40/74 (54)
AVs (both directions)	14/74 (19)
AV/VA and AA	3/74 (4)
AV/VA and VV	2/74 (3)
Only AA	2/74 (3)
Only VV	2/74 (3)
AV/VA, AA and VV	1/74 (1)
All anastomoses diameter < 1 mm	62/64 (97)

Data are the median (IQR) or n/N (%). TAPS, twin anemia polycythemia; TTTS, twin-twin transfusion syndrome; AV, arterio-venous; VA, veno-arterial; AA, arterio-arterial; VV, veno-venous.

**Table 4:** Perinatal outcome in the post-laser twin anemia polycythemia sequence.

	Post-Laser TAPS (*N* = 164 Pregnancies; 328 Fetuses)	TAPS Donors (*N* = 164 Fetuses)	TAPS Recipients (*N* = 164 Fetuses) †	*p*-Value
GA at birth	31.7 (28.6–33.7; 19.0–41.3)			
Fetal demise †	56/327 (17)	43/164 (26)	13/163 (8)	<0.001
Spontaneous	33/327 (10)	25/164 (15)	8/163 (5)	<0.001
Intended	23/327 (7)	18/164 (11)	5/163 (3)	0.007
Neonatal mortality	27/271 (10)	18/121 (15)	9/150 (6)	0.008
Perinatal mortality (overall) †	83/327 (25)	61/164 (37)	22/163 (14)	<0.001
Perinatal mortality (spontaneous) †	60/327 (18)	43/164 (26)	17/163 (10)	<0.001
Severe neonatal morbidity ‡	105/263 (40)	51/118 (43)	54/145 (37)	0.568
Respiratory distress syndrome	88/263(34)	42/118 (36)	46/145 (32)	0.945
Patent ductus arteriosus	20/263 (8)	11/118 (9)	9/145 (6)	0.278
Necrotizing enterocolitis	6/263 (2)	4/118 (3)	2/145 (1)	0.275
Retinopathy of prematurity	9/263 (3)	6/118 (5)	3/145 (2)	0.165
Amniotic band syndrome	3/263 (1)	2/118 (2)	1/145 (1)	0.451
Severe cerebral injury	28/263 (11)	13/118 (11)	15/145 (10)	0.916
Birth weight (g) §	1390 ± 567	1346 ± 525	1533 ± 588	<0.001
Severe growth restriction (bw < p3) §	62/270 (23)	31/121 (26)	31/149 (21)	0.267
Mild growth restriction (bw < p10) §	122/270 (45)	60/121 (50)	61/149 (41)	0.061

Data are presented as the means ± SD, medians (IQR), or n/N (%). † One missing value (1 post-laser TAPS recipient with unknown perinatal outcome). ‡ Nine missing values (same as “†” plus 4 cases with unknown neonatal morbidity info and 4 cases that died shortly after birth). § Two missing values (same as “†” plus one case with missing birth weight). TAPS, twin anemia polycythemia sequence; GA, gestational age; bw, birth weight.

## Appendix A

**Table A1:** Participating fetal therapy centers and their number of contributed post-laser TAPS cases.

Center	Country	Post-Laser TAPS Cases
Leiden University Medical Center	The Netherlands	66
Children’s Hospital V. Buzzi Milan	Italy	17
Necker-Enfants Malades Hospital Paris	France	16
Leuven University Hospital	Belgium	13
Center Medico-Chirurgical Obstetrical Strasbourg	France	10
Mount Sinai Hospital Toronto	Canada	10
Saint George’s Hospital London	United Kingdom	5
Birmingham Women’s and Children’s NHS Foundation Trust	United Kingdom	5
Medical University of Graz	Austria	3
Mater Hospital Brisbane	Australia	4
University of Texas McGovern Medical School at Houston	United States of America	4
V.I. Kulakov National Medical Research Center of Obstetrics, Gynecology and Perinatology Moscow	Russia	4
Karolinska University Hospital Stockholm	Sweden	3
Hospital Universitari Vall d’Hebron Barcelona	Spain	2
Brugmann University Hospital	Belgium	1
University Medical Center Hamburg-Eppendorf	Germany	1

## Appendix B

**Table A2:** Univariate and multivariate risk analysis for spontaneous perinatal mortality in the post-laser twin anemia polycythemia sequence.

	Death † (*n* = 60/304)	Alive † (*n* = 244/304)	Univariate Analysis OR (95% CI)	SE	*p*	Multivariate Analysis OR (95% CI)	SE	*p*
GA at diagnosis TAPS	23.3 ± 3.3	23.7 ± 3.7	1.0 (0.9–1.1)	0.1	0.623			
GA at laser TTTS	20.1 ± 2.4	20.8 ± 3.3	0.9 (0.8–1.0)	0.1	0.233			
Days between TTTS laser and TAPS	20.9 ± 18.4	21.8 ± 23.1	1.0 (1.0–1.0)	0.0	0.829			
Quintero stage for TTTS ‡								
1	3/35 (9)	32/35 (91)	- *			- *		
2	27/140 (19)	113/140 (81)	2.5 (0.7–8.5)	0.6	0.139	1.7 (0.4–6.9)	0.7	0.439
3	27/111 (24)	84/111(76)	3.6 (1.1–12.0)	0.6	0.039	3.5 (0.9–13.2)	0.7	0.069
4	2/10 (20)	8/10 (80)	2.8 (0.2–32.4)	1.3	0.418	3.4 (0.3–33.6)	1.2	0.298
Antenatal TAPS stage								
1	6/47 (13)	41/47 (87)	- *			- *		
2	12/122 (10)	110/122 (90)	0.9 (0.3–3.2)	0.6	0.864	0.8 (0.2–3.2)	0.7	0.790
3	14/56 (25)	50/56 (75)	2.6 (0.7–9.1)	0.6	0.136	2.6 (0.6–10.3)	0.7	0.182
4	20/46 (44)	26/46 (55)	6.1 (1.8–20.4)	0.6	0.003	6.1 (1.4–26.0)	0.7	0.015
TAPS recipient	17/158 (11)	146/158 (89)	- *			- *		
TAPS donor	43/146 (30)	103/146 (70)	3.7 (2.2–6.3)	0.3	<0.001	4.2 (2.1–8.3)	0.3	<0.001
Persistence of TTTS-TAPS donor-role, no §	25/162 (15)	137/162 (85)	- *					
Persistence of TTTS-TAPS donor-role, yes §	34/137 (25)	103/137 (77)	1.8 (1.0–3.5)	0.3	0.061			
Antenatal therapy								
Expectant management	27/124 (22)	97/124 (78)	- *					
Delivery	4/18 (22)	14/18 (78)	1.4 (0.3–4.2)	0.6	0.622			
IUT (± PET)	16/81 (20)	72/81 (82)	0.9 (0.4–1.9)	0.4	0.893			
Laser surgery	9/44 (21)	38/44 (79)	1.0 (0.3–2.8)	0.5	0.942			
Selective feticide (co-twin)	0/11 (0)	11/11 (100)	0.3 (0.0–2.9)	1.1	0.330			
GA at birth	29.5 ± 5.0	31.6 ± 3.1	0.8 (0.7–1.0)	0.1	0.006	0.8 (0.7–0.9)	0.1	0.001

Values are the odds ratios (OR) (95% confidence intervals (CI)), the standard error (SE), and the *p*-value. * Set to zero as the reference. † Twenty-three of 327 cases were excluded based on selective feticide or termination of pregnancy. ‡ Five cases had a missing Quintero stage value.§ In 3 cases, persistence of TTS-TAPS donor-role was unknown. ¶ SPSS was not able to calculate OR for groups in which the event (spontaneous mortality) did not occur; therefore, a correction for non-occurring events was applied, and with this correction, an unaffected child was changed into an affected child, for all groups. TAPS, twin anemia polycythemia sequence; GA, gestational age; TTTS, twin-twin transfusion syndrome; IUT, intrauterine transfusion; PET, partial exchange transfusion.

**Table A3:** Univariate and multivariate risk analysis for spontaneous perinatal mortality in the post-laser twin anemia polycythemia sequence.

	SNM † (139/429)	No SNM † (290/429)	Univariate Analysis OR (95% CI)	SE	*p*	Multivariate Analysis OR (95% CI)	SE	*p*
GA at diagnosis TAPS	24.5 ± 3.1	23.4 ± 4.0	0.9 (0.8–1.0)	0.1	0.090			
GA at laser TTTS	21.1 ± 2.6	20.6 ± 3.6	0.9 (0.8–1.0)	0.1	0.261			
Days between TTTS laser and TAPS	22.3 ± 21.9	22.1 ± 23.9	1.0 (1.0–1.0)	0.0	0.987			
Quintero stage for TTTS								
1	8/34 (24)	26/34 (76)	- *			- *		
2	64/123 (52)	59/123 (48)	3.2 (1.1–9.7)	0.6	0.038	2.8 (0.9–9.0)	0.6	0.066
3	28/89 (32)	61/89 (68)	1.3 (0.4–4.2)	0.6	0.617	1.3 (0.4–3.9)	0.6	0.690
4	3/10 (30)	3/10 (70)	1.3 (0.2–9.3)	1.0	0.782	1.1 (0.1–10.0)	1.1	0.959
Antenatal TAPS stage								
1	18/45 (40)	30/45 (60)	- *					
2	41/112 (37)	71/112 (63)	1.0 (0.4–2.5)	0.5	0.970			
3	16/45 (36)	29/45 (64)	0.7 (0.2–2.2)	0.6	0.570			
4	17/32 (53)	15/32 (47)	1.6 (0.5–5.0)	0.6	0.381			
TAPS recipient	54/145(37)	91/145 (63)	- *					
TAPS donor	51/118 (43)	67/118 (57)	1.1 (0.8–1.5)	0.2	0.568			
Persistence of TTTS-TAPS donor-role, no	47/139 (34)	92/139 (66)	- *					
Persistence of TTTS-TAPS donor-role, yes	58/120 (48)	62/120 (52)	1.6 (0.9–3.0)	0.3	0.116			
Antenatal therapy								
Expectant management	34/100 (34)	66/100 (66)	- *					
Delivery	9/17 (53)	8/17 (47)	2.1 (0.6–8.1)	0.7	0.270			
IUT (± PET)	34/72 (47)	38/72 (53)	1.7 (0.8–3.7)	0.4	0.158			
Laser surgery	12/37 (32)	25/37 (68)	1.1 (0.4–2.9)	0.5	0.851			
Selective feticide	3/11 (27)	8/11 (73)	0.7 (0.2–3.2)	0.7	0.720			
GA at birth	29.4 ± 2.6	32.9 ± 2.8	1.5 (1.3–1.7)	0.1	<0.001	1.5 (1.3–1.7)	0.1	<0.001
Severe growth restriction, no	81/200 (41)	119/200 (60)	- *					
Severe growth restriction, yes	24/62 (39)	38/62 (61)	1.3 (0.8–2.2)	0.3	0.351			
Postnatal TAPS, no	23/58 (40)	35/58 (60)	- *					
Postnatal TAPS, yes	64/151 (42)	87/151(58)	1.1 (0.5–2.5)	0.4	0.800			

Values are the odds ratios (OR) (95% confidence intervals (CI)), the standard error (SE), and the *p*-value. * Set to zero as the reference. † Nine missing values (6 cases with unknown neonatal outcome and 3 neonates that died shortly after birth). SNM, severe neonatal morbidity; TAPS, twin anemia polycythemia sequence; GA, gestational age; IUT, intrauterine transfusion; PET, partial exchange transfusion.
